# Long-Term Postsurgical Outcomes of Neoadjuvant Chemoradiation (CROSS) Versus Chemotherapy (FLOT) for Multimodal Treatment of Adenocarcinoma of the Esophagus and the Esophagogastric Junction

**DOI:** 10.1245/s10434-023-13643-9

**Published:** 2023-05-21

**Authors:** Florian Gebauer, Patrick S. Plum, Alexander Damanakis, Seung-Hun Chon, Felix Popp, Thomas Zander, Alexander Quaas, Hans Fuchs, Thomas Schmidt, Wolfgang Schröder, Christiane J. Bruns

**Affiliations:** 1 Department of General, Visceral, Cancer and Transplantation Surgery, Faculty of Medicine, University Hospital Cologne, Cologne, Germany; 2 Gastrointestinal Cancer Group Cologne (GCGC), Cologne, Germany; 3 Department of General and Visceral Surgery, Helios University Hospital of Wuppertal, Wuppertal, Germany; 4 Department of Visceral, Transplant, Thoracic and Vascular Surgery, University Hospital Leipzig, Leipzig, Germany; 5 Department I of Internal Medicine, Faculty of Medicine, Center for Integrated Oncology (CIO), University Hospital Cologne, Cologne, Germany; 6 Institute of Pathology, Faculty of Medicine, University Hospital Cologne, Cologne, Germany

**Keywords:** Multimodal treatment, Neoadjuvant radiochemotherapy, Perioperative chemotherapy, CROSS, FLOT, Long-term prognosis, Esophageal adenocarcinoma

## Abstract

**Background:**

The question of the ideal neoadjuvant therapy for locally advanced esophagogastric adenocarcinoma has not been answered to date. Multimodal treatment has become a standard treatment for these adenocarcinomas. Currently, perioperative chemotherapy (FLOT) or neoadjuvant chemoradiation (CROSS) is recommended.

**Methods:**

A monocentric retrospective analysis compared long-term survival after CROSS versus FLOT. The study enrolled patients with adenocarcinoma of the esophagus (EAC) or the esophagogastric junction type I or II undergoing oncologic Ivor–Lewis esophagectomy between January 2012 and December 2019. The primary objective was to determine the long-term outcome in terms of overall survival. The secondary objectives were to determine differences regarding the histopathologic categories after neoadjuvant treatment and the histomorphologic regression.

**Results:**

The findings showed no survival advantage for one or the other treatment in this highly standardized cohort. All the patients underwent open (CROSS: 9.4% vs. FLOT: 22%), hybrid (CROSS: 82% vs. FLOT: 72%), or minimally invasive (CROSS: 8.9% vs. FLOT: 5.6%) thoracoabdominal esophagectomy. The median post-surgical follow-up period was 57.6 months (95% confidence interval [CI] 23.2–109.7 months), and the median survival was longer for the CROSS patients (54 months) than for the FLOT patients (37.2 months) (*p* = 0.053). The overall 5-years survival was 47% for the entire cohort (48% for the CROSS and 43% for the FLOT patients). The CROSS patients showed a better pathologic response and fewer advanced tumor stages.

**Conclusion:**

The improved pathologic response after CROSS cannot be translated into longer overall survival. To date, the choice of which neoadjuvant treatment to use can be made only on the basis of clinical parameters and the patient’s performance status.

Adenocarcinoma of the esophagus is one of the tumor entities with the highest mortality of all gastrointestinal tumors worldwide. Its incidence in the Western industrialized nations has increased significantly in recent years.^1–5^

In the majority of cases, esophageal cancer is diagnosed at a locally advanced stage. Currently, for advanced tumors and/or evidence of lymph node metastases (c/uT2N+; >c/uT3N0, respectively, N+), neoadjuvant therapy in the context of a multimodal therapy regimen is recommended by national and international guidelines. In 2006, the MAGIC study was the first to demonstrate the superiority of perioperative chemotherapy in the treatment of gastric cancer with regard to overall survival (OS).^6^ Subsequently, the concept of multimodal therapy for adenocarcinomas of the esophagus and the gastroesophageal junction (GEJ) types I to III has been incorporated into treatment guidelines from major international cancer societies such as the European Society of Medical Oncology (ESMO) and the National Comprehensive Cancer Network (NCCN).

Two distinctly different strategies have evolved: perioperative chemotherapy (CT) and neoadjuvant chemoradiation (CRT). Perioperative chemotherapy alone has shown a better local response, with significantly better tolerability and fewer toxicity events due to the implementation of new chemotherapeutic regimens (FLOT comprising 5-fluorouracil/leucovorin, oxaliplatin, docetaxel).^7^ In parallel, a Dutch research group showed a survival benefit after neoadjuvant chemoradiotherapy in the CROSS trial. For esophageal adenocarcinomas, the OS was improved by 7% with the CROSS protocol consisting of CRT with 41 Gy, carboplatin, and paclitaxel.^8^

However, the best therapeutic concept of multimodal therapy for patients with adenocarcinomas of the esophagus or the gastroesophageal junction still is not conclusively clarified. The choice of chemotherapy or chemoradiotherapy often is based on the personal preference of the treating physician, institutional constraints, or geographic conditions.

No direct comparison of the two strategies is available, and the primary end point (OS) of the ESOPEC trial (NCT02509286) comparing CROSS and FLOT for GEJ types I to III adenocarcinomas will not be available before the end of 2023.^9^ Recently published preliminary data from the Neo-AEGIS trial comparing different perioperative chemotherapies (ECF/ECX/FLOT [ECF/ECX/EOF/EOX pre-2018, FLOT option 2019/20]) with neoadjuvant CRT according to the CROSS regimen showed no clear evidence that one or the other procedure was superior in terms of tumor recurrence.^10,11^ The primary outcome parameter was the OS at the end of the trial with up to 3-years of follow-up evaluation, but the data have not been reported to date.

The current study compared chemotherapy (FLOT) and CRT (CROSS) for malignant tumors of the upper gastrointestinal tract in terms of long-term oncologic outcomes after neoadjuvant therapy in a large monocentric patient population from a nationwide tertiary medical center. All the patients received highly standardized oncologic surgery (Ivor–Lewis esophagectomy and two-field lymphadenectomy) and experienced adenocarcinoma of either the esophagus or GEJ type I or II.

## Methods and Materials

### Study Cohort and Settings

This study was a monocentric analysis of a nationwide high-volume center for upper gastrointestinal tumors. Between January 2012 and December 2019, 1068 patients underwent elective esophagectomy due to malignant diseases of the esophagus or the esophagogastric junction at the Department of General, Visceral, Cancer and Transplantation Surgery, University Hospital of Cologne. All the patients received primary staging consisting of esophagogastroduodenoscopy with biopsy, endoscopic ultrasound, and spiral contrast-enhanced computer tomography of the thorax and abdomen, and then were discussed in a multidisciplinary tumor conference to determine the best therapeutic options. The study excluded patients with histologic subtypes other than adenocarcinoma (EAC or GEJ I or II), and only patients who qualified for multimodal treatment before surgical resection (cT2-4 and/or cN+) were considered for further analysis. Patients with an indication of primary resection or patients who received chemotherapeutic regimens other than FLOT also were excluded from the current study.

Follow-up data were collected during postoperative cancer surveillance visits by direct contact with the patient, by mail, by telephone, by contact with the general practitioner, or by consultation of the central civil registers. The analysis was performed according to the guidelines of the Institutional Ethics Committee of the University Hospital of Cologne.

### Neoadjuvant Treatment Regimens

All the patients enrolled in the study either had neoadjuvant CRT analogous to the CROSS regimen including 41.4 Gy radiation, carboplatin, and paclitaxel, or underwent neoadjuvant chemotherapy analogous to the FLOT regimen with 5-fluorouracil/leucovorin, oxaliplatin, and docetaxel according to the original clinical trials published in 2012 respectively in 2008.^7,8^

### Surgical Resection

The standard surgical procedure was two-stage Ivor–Lewis esophagectomy with gastrolysis and right transthoracic en bloc esophagectomy including two-field lymphadenectomy of mediastinal and abdominal lymph nodes. Reconstruction was performed by high intrathoracic esophagogastrostomy, as described previously.^12–15^ Surgery was performed via open access approaches or hybrid resections (laparoscopic gastrolysis followed by open thoracotomy), or as totally minimally invasive procedures. Other surgical approaches such as three-stage McKeown esophagectomy and transhiatal gastrectomy were excluded in this study.

### Histopathologic Evaluation

Resected specimens and harvested lymph nodes were fixed within 5% formaldehyde and embedded in paraffin before staining. Histopathologic analysis and classification were performed by an experienced gastrointestinal pathologist according to the eighth edition of the Union for International Cancer Control (UICC)/TNM-classification of malignant tumors including tumor location, depth of tumor infiltration, grading, residual tumor, and total number of resected and infiltrated lymph nodes.^16^

The degree of histomorphologic regression after neoadjuvant therapy was classified into four categories according to the Cologne Regression Scale:^17^ grade 1 (> 50% vital residual tumor cells), grade 2 (10% to 50% vital residual tumor cells), grade 3 (a nearly complete response and < 10% vital residual tumor cells) and grade 4 (complete response excluding any vital tumor remains). Additionally, post-neoadjuvant therapy (ypTNM) stage groups were summarized in concordance with Rice et al.^18^ to guarantee further comparability.

### Statistical Analysis

The current study aimed primarily at the long-term outcome in terms of the OS. Overall survival was defined as the time from surgery to death or the date of the last follow-up visit (censored patient data). The secondary objectives were differences regarding the histopathologic categories after neoadjuvant treatment and degree of histomorphologic regression toward neoadjuvant therapy. Other outcomes such as the 90-days mortality rate, the rate of postsurgical complications according to Clavian-Dindo, and functional differences (e.g., pylorospasm with consecutive delayed gastric emptying) have already been analyzed and published recently.^12^

Survival data were analyzed using the log-rank test, and the survival curves were established based on the Kaplan-Meier method. Regression analysis was performed according to the Cox proportional hazard model to calculate hazard ratios (HRs) together with their 95% confidence intervals (CIs). Continuous variables were compared using the Kruskall-Wallis rank-sum test, and categorical variables were compared according to Pearson’s chi-square test where applicable. In all analyses, a two-sided *p* value lower than 0.05 was considered statistically significant. Data were analyzed using R version 4.1.2 inside the R studio for Apple Mac.

## Results

### Clinical Characteristics of the Patients

After application of the pre-defined exclusion criteria (histology other than adenocarcinoma of the esophagus or GEJ, primary resection, inoperability) to the 1068 patients receiving elective esophagectomy between January 2012 and December 2019, 578 patients received either CROSS (*n* = 416, 72.0%) or FLOT (*n* = 162, 18%) with neoadjuvant intention and therefore remained for further analysis. The clinicopathologic baseline characteristics of the cohort are presented in Table 1.

**Table 1:** Clinicopathologic characteristics of patients with esophageal adenocarcinoma who underwent multimodal treatment with either neoadjuvant CROSS or FLOT between 2012 and 2019

Characteristic	Clinical data of the study cohort after neoadjuvant chemotherapy according to CROSS or FLOT			*p* value^a^
	Overall (*n* = 578) *n* (%)	CROSS (*n* = 416) *n* (%)	FLOT (*n* = 162) *n* (%)	
Histology				
EAC	578 (100)	416 (100)	162 (100)	
Mean age: years (min–max)	61 ± 10 (27–85)	61 ± 10 (27–83)	61 ± 10 (33–85)	0.800
Sex				0.528
Female	76 (13)	57 (14)	19 (12)	
Male	502 (87)	359 (86)	143 (88)	
Mean BMI: kg/m^2^ (min–max)	26.3 ± 4.5 (14.5–45.1)	26.0 ± 4.5 (14.5–45.1)	27.2 ± 4.5 (17.7–41.8)	0.009
Procedure				< 0.001
Hybrid	457 (79)	340 (82)	117 (72)	
Open surgery	75 (13)	39 (9.4)	36 (22)	
Total minimally invasive	46 (8.0)	37 (8.9)	9 (5.6)	
pT-category				0.002
pT0	122 (21)	95 (23)	27 (17)	
pT1	97 (17)	72 (17)	25 (15)	
pT2	87 (15)	65 (16)	22 (14)	
pT3	259 (45)	181 (44)	78 (48)	
4	13 (2.2)	3 (0.7)	10 (6.2)	
pN-category				< 0.001
pN0	321 (56)	251 (60)	70 (43)	
pN1	112 (19)	72 (17)	40 (25)	
pN2	82 (14)	61 (15)	21 (13)	
pN3	63 (11)	32 (7.7)	31 (19)	
Cologne Regression Scale (local response)				< 0.001
I (complete response)	108 (19)	83 (21)	25 (16)	
II (< 10% VTCs)	151 (27)	120 (30)	31 (20)	
III (10–50% VTCs)	168 (30)	124 (31)	44 (28)	
IV (>50% VTCs)	132 (24)	77 (19)	55 (35)	

*EAC* esophageal adenocarcinoma, *Min* minimum, *Max* maximum, *BMI* body mass index, *VTCs* vital tumor cells

^a^Wilcoxon rank-sum test; Pearson’s chi-square test; Fisher’s exact test

As expected for esophageal cancer, the male sex was predominant in both groups (86% of the CROSS patients and 88% of the FLOT patients; *p* = 0.528). The median age was 61-years for both subgroups (CROSS [range, 27–83 years] vs. FLOT [range, 33–85 years]; *p* = 0.800). The median body mass index (BMI) was 27.2 kg/m^2^ (range, 17.7–41.8 kg/m^2^) among the FLOT regimen patients, which was significantly higher than the median BMI (26.0 kg/m^2^; range, 14.5–45.1 kg/m^2^) among the CROSS treatment group (*p* = 0.009). Significantly more patients in the CROSS cohort underwent either hybrid (CROSS 82% vs. FLOT 72%) or minimally invasive (CROSS 8.9% vs. FLOT 5.6%) procedures, whereas more patients of the FLOT subgroup had open surgical approaches (CROSS 9.4% vs. FLOT 22%; *p* < 0.001).

### Primary Objective

*Oncologic Long-Term Prognosis and Post-Neoadjuvant Therapy Stage Groups.* The median post-surgical follow-up period was 57.6 months (95% CI 23.2–109.7 months). The median follow-up period for neoadjuvant CRT was 62.1 months (minimum 23.6 months–maximum 106.5 months), whereas the median follow-up period for induction chemotherapy was 47.8 months (minimum 23.2 months–maximum 109.7 months).

**Fig. 1:**
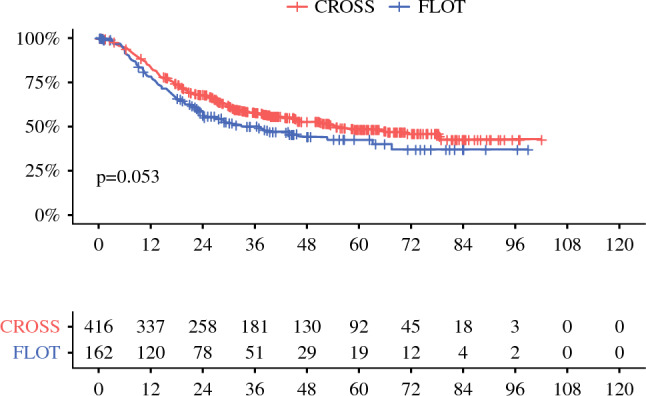
Kaplan–Meier survival analysis (log-rank test) of the long-term survival (in months) of patients who received neoadjuvant CROSS versus FLOT followed by esophagectomy due to esophageal adenocarcinoma (*n* = 578). Patients receiving neoadjuvant chemoradiation analogous to the CROSS regimen showed a (non-significantly) better (*p* = 0.053) postsurgical outcome than those who received neoadjuvant chemotherapy analogous to the FLOT regimen.

Survival analysis for the entire and unbalanced patient cohort did not show any survival difference, although it should be noted that the significance level of 0.05 was just missed, and the median survival was longer among the CROSS patients than among the FLOT patients (54 vs. 37.2 months; *p* = 0.053). The overall 5-years survival rate was 47% for the entire cohort (48% for the patients receiving CROSS and 43% for the patients undergoing FLOT). The details are illustrated in Table 2 and Fig. 1.

**Table 2:** Median and 5-year survival of the cohort with esophageal adenocarcinoma depending on the neoadjuvant therapy (CROSS vs. FLOT)

Characteristic	Median and 5-years survival rates in the entire cohort			
	Survival probability		Survival time	
	*n*	5-Years survival % (95% CI)	Median survival Months (95% CI)	*p* value^a^
Overall	578	47 (42–51)		
Neoadjuvant therapy	578			0.053
CROSS		48 (43–54)	54.0 (44.4–NA)	
FLOT		43 (34–53)	37.2 (24.0–67.2)	

*CI* confidence interval

^a^Log-rank test

Additionally, subgroup analyses of the different residual infiltration depths (ypT) and locoregional lymphatic metastases (ypN) were performed separately. The outcome was comparable between the ypT0 and ypT1 patients after multimodal treatment with either CROSS or FLOT (5-years survival for ypT0: CROSS 71% vs. FLOT 61%; *p* = 0.806). The median survival was not reached in either group (5-years survival for ypT1: CROSS 58% vs. FLOT 50% [*p* = 0.271]; median survival for FLOT 55.2 months vs. CROSS not reached).

Similarly, the patients with ypT2 showed non-significant prognostic differences (5-years survival for ypT2: CROSS 34% vs. FLOT 57% [*p* = 0.177]; median survival for CROSS 33.6 months vs. FLOT not reached). The prognosis for the ypT3 patients was favorable for neoadjuvant CRT, but the outcomes did not differ significantly (5-years survival for ypT3: CROSS 36% vs. FLOT 32% [*p* = 0.076]; median survival: CROSS 31.2 months vs. FLOT 22.8 months). None of the patients with ypT4 tumors were alive 5-years after surgery in either group (*p* = 0.770) (median survival: CROSS 16.8 months vs. FLOT 22.8 months).

**Fig. 2:**
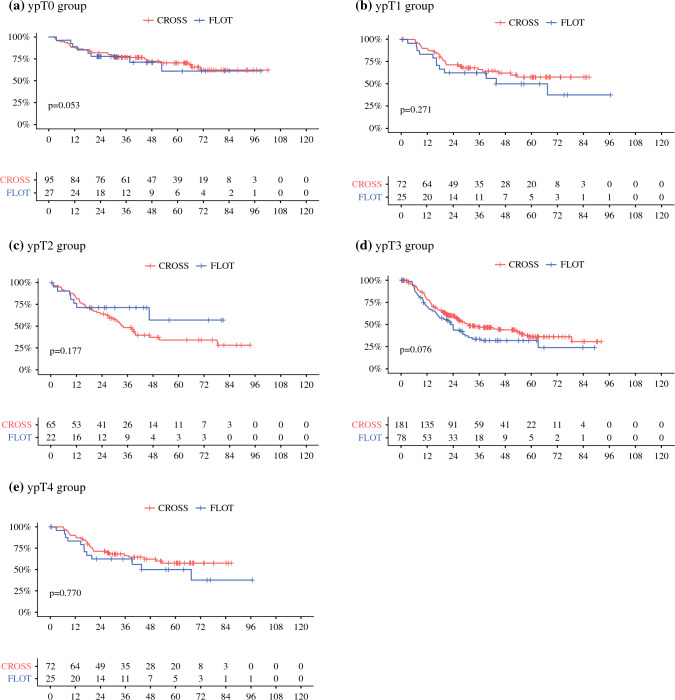

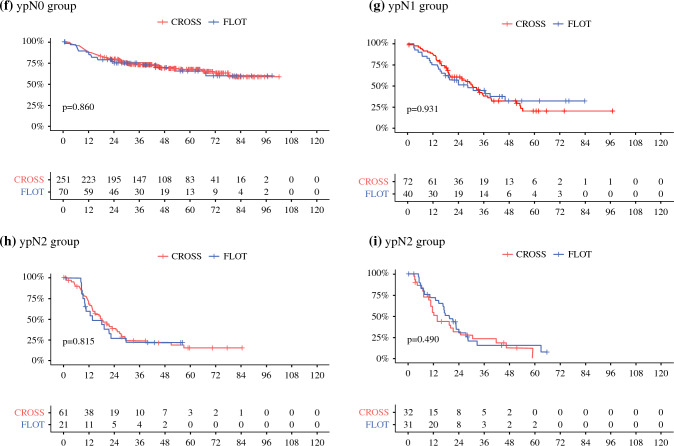
Kaplan–Meier survival analysis (log-rank test) of the patients (in months) depending on the resulting ypT and ypN status after neoadjuvant CROSS versus FLOT followed by esophagectomy due to esophageal adenocarcinoma. Subgroup survival analyses of the cohort according to the respective ypT (**a–e**) and ypN (**f–i**) status of the patients. No prognostically significant difference could be found for all the different groups. The majority of the patients showed an only limited therapeutic response (ypT3). In this context, (**d**) neoadjuvant chemoradiation analogous to the CROSS regimen had a survival benefit compared with neoadjuvant chemotherapy analogous to the FLOT protocol (although statistical significance was not reached; *p* = 0.076). Similarly, the prognosis was (non-significantly) better for the ypT1 patients (**b**) in the CROSS versus the FLOT cohort.

Comparison of the resulting ypN status showed no significant alterations depending on the kind of neoadjuvant therapeutic concept. The patients with no lymph node metastasis after multimodal treatment (ypN0) did not differ between CROSS and FLOT (5-years survival for ypT0: CROSS 67% vs. FLOT 66%; *p* = 0.860). Median survival was not reached in either group, and the subgroup analyses showed no prognostic benefit for either the ypN1 patients (5-years survival: CROSS 21% vs. FLOT 32% [*p* = 0.931]; median survival: CROSS 30 months vs. FLOT 28.8 months) or the ypN2 patients (5-years survival: CROSS 15% vs. FLOT 0% [*p* = 0.815]; median survival: CROSS 18 months vs. FLOT 13.2 months). No patients with ypN3 survived 5-years after CROSS, whereas the 5-years survival rate was 16% among the FLOT patients (*p* = 0.490) (median survival was not reached for either group, data not shown). Figure 2 presents further details.

Post-neoadjuvant therapy stages (ypTNM) also were grouped according to UICC classification, as described by Rice et al.^18^, and putative survival differences in the subgroups depending on the neoadjuvant treatment were compared. No significant prognostic variations were found in those post-neoadjuvant therapy stages from early UICC groups to locally advanced UICC tumors.

**Fig. 3:**
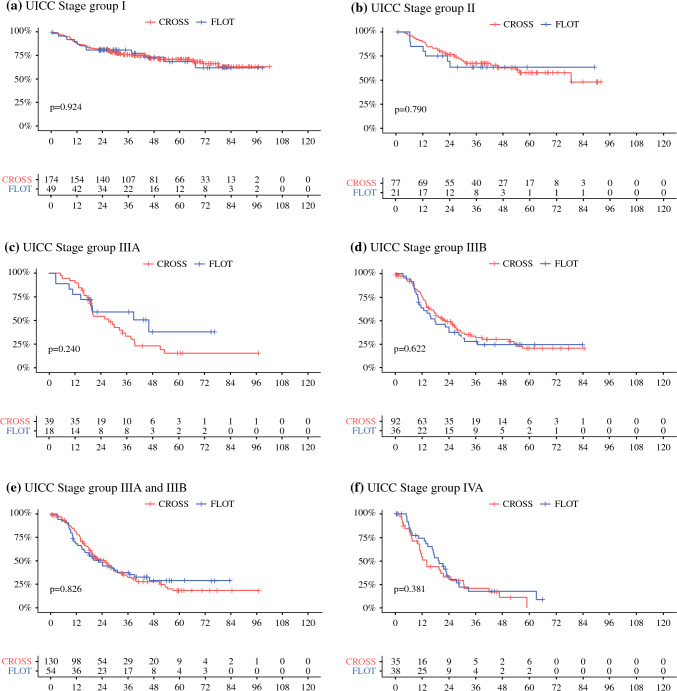
Kaplan–Meier survival analysis (log-rank test) of the patients depending on the post-neoadjuvant therapy (ypTNM) stage groups according to UICC after neoadjuvant CROSS versus FLOT followed by esophagectomy due to esophageal adenocarcinoma. UICC, Union for International Cancer Control. Subgroup survival analyses of the cohort according to post-neoadjuvant therapy (ypTNM) stage groups as shown by UICC. (**a–f)** No significant prognostic differences in dependence are shown in CROSS versus FLOT. UICC, Union for International Cancer Control

Among early UICC stage I patients, 174 CROSS and 49 FLOT patients were compared, without survival benefits depending on the kind of neoadjuvant treatment (*p* = 0.924). Subgroup analysis of the 77 patients with CROSS versus the 21 patients with FLOT among the UICC stage II patients also did not show significant prognostic differences (*p* = 0.790). The prognoses of the patients with locally advanced UICC stage II tumors (CROSS 39 vs. FLOT 18) or stage III tumors (CROSS 91 vs. FLOT 36) exhibited no superiority of any neoadjuvant therapeutic concept in these groups (*p* = 0.240 vs. 0.622). There also were no relevant prognostic differences in the subgroup of UICC stage IV patients (35 patients after CROSS vs. 38 patients after FLOT; *p* = 0.381). Figure 3 summarizes more details on the Kaplan-Meier survival analyses performed.

Applying uni- and multivariate analyses to the entire patient cohort, including parameters such as the neoadjuvant treatment concept, the UICC by ypTNMN, the gender, or the pN-status showed that the type of neoadjuvant therapy was not an independent factor for prognosis of the patients (*p* = 0.058 vs. 0.831) in either analysis. In contrast this, the pN status was identified as an independent parameter in both the uni- and multivariate COX regressions (*p* < 0.001)). The post-surgical UICC staging showed stages IIIA to IVA to be significant parameters in the univariate analysis (each *p* < 0.001, data not shown), whereas in the multivariate analysis, only stage IIIA disease reached the significance level (*p* = 0.009; Table 3).

**Table 3:** Cox proportional hazards analysis of independent prognostic factors

Characteristic	Univariable COX regression					Multivariable COX regression			
	*n*	HR	95% CI	*p* value	*q* value^a^	HR	95% CI	*p* value	*q* value^a^
ypT	578			< 0.001	< 0.001			0.341	> 0.999
0		–	–			–	–		
1		1.52	0.97–2.39			1.32	0.84–2.07		
2		2.24	1.45–3.46			1.49	0.95–2.34		
3		2.76	1.92–3.96			1.48	0.99–2.21		
4		4.84	2.24–10.4			1.73	0.77–3.93		
ypN	578			< 0.001	< 0.001			< 0.001	< 0.001
0		–	–			–	–		
1		2.79	2.05–3.79			2.57	1.86–3.54		
2		4.37	3.15–6.06			3.79	2.65–5.41		
3		5.12	3.60–7.29			4.43	2.97–6.62		
Neoadjuvant therapy	578			0.058	0.174			0.831	> 0.999
CROSS		–	–			–	–		
FLOT		1.29	1.00–1.67			0.97	0.74–1.28		

*HR* hazard ratio, *CI* confidence interval

^a^Bonferroni correction for multiple testing

### Secondary Objectives

*Histopathologic Results and Pathologic Response.* The histopathologic results of the resected specimens showed a higher proportion of patients with locally advanced tumor stages including ypT4 (CROSS 0.7% vs. FLOT 6.2%) and ypN3 (CROSS 7.7% vs. FLOT 19%) among the FLOT patients (*p* = 0.002 vs. *p* < 0.001). On the other hand, 122 patients (21.1%) of the entire unbalanced cohort showed a complete pathologic response (cpR) with no viable malignant cells in the primary tumors (ypT0) or harvested lymph nodes (ypN0). This frequency of ypT0 status was higher in the CROSS group (*n* = 95, 23%) than in the FLOT group (*n* = 27, 17%) (*p* = 0.002). Similarly, in the subgroup analysis, 321 (56%) of the patients in the entire cohort presented with ypN0 status (251 CROSS patients [60%] vs. 70 FLOT patients [43%]; *p* < 0.001). Therefore, a complete pathologic response (cpR) was higher in CROSS cohort than in the FLOT cohort. More details are presented in Table 1.

The histopathologic response rate of the primary tumor also was evaluated according to the so-called Cologne Regression Scale (CRS) reflecting the percentage of vital tumor cells, as published previously.^17^ The regression rate differed significantly depending on the type of neoadjuvant treatment, resulting in a higher proportion of patients after neoadjuvant FLOT who still showed large amounts of viable tumor cells (VCTs) in the resected specimens (Cologne Regression Scale IV for 77 CROSS patients [10%] vs. 55 FLOT patients [35%]; *p* < 0.001). On the other hand, Cologne Regression Scale I was reached by more patients in the CROSS cohort (*n* = 83, 21%) than in the FLOT cohort (*n* = 25, 16%).

## Discussion

The current study investigated the impact of multimodal therapeutic concepts on OS in adenocarcinoma of the esophagus and GEJ types I and II. Since the results of the MAGIC, FLOT4-AIO, and CROSS trials, peri- and preoperative therapy concepts with proven evidence for improved OS have been integrated into the treatment guidelines for esophageal cancer.^6–8^ Nevertheless, the question concerning superiority of either perioperative chemotherapy or preoperative radiochemotherapy alone has not been conclusively answered.

This study aimed to evaluate oncologic outcomes in the setting of routine patient care at a tertiary high-volume center for esophageal cancer surgery and to identify potential differences in OS after neoadjuvant CROSS versus FLOT therapy. However, the study could not show a clear survival advantage for one or the other procedure. This is concordant with our previous analysis examining putative postsurgical differences regarding 30- versus 90-days mortality or morbidity in EAC patients who underwent esophagectomy after either neoadjuvant CROSS or FLOT treatment.^12^ Both concepts showed non-superiority in short-term outcome and were not associated with severe post-surgical complications.

Regarding the end point of OS, we did not identify a significant difference between the CROSS and FLOT groups, but the FLOT patients showed a tendency for a shorter survival, although this difference did not reach the significance level in the statistical analyses. In our study, we found a 5-years survival rate of 47% in the CRT group and 43% in the chemotherapy group.

The data we have shown are relevant in several aspects. The comparison between neoadjuvant CRT and perioperative chemotherapy has already been investigated in several studies.^11,19–33^ Most of the studies published to date also have failed to detect a difference between neoadjuvant radiochemotherapy and perioperative chemotherapy. However, the majority of studies had a very small number of cases,^19–22^ different tumor types (with deviating histologies including both EAC and esophageal squamous cell carcinoma [ESCC]) studied in combination,^23–26^ a chemotherapeutic regimen not homogeneous within the cohorts,^11,27–29^ and surgical operation procedures that were not comparable.^29–33^

A strength and novelty of the current study was the very homogeneous patient population. For the current study, we intentionally included only patients treated according to either the CROSS or FLOT protocol. No deviating chemo- or radiochemotherapy protocols were considered for the analysis, which enabled a comparison of the two groups with only minimal bias in the administrated therapy. In addition, only patients with esophageal adenocarcinoma and GEJ (Siewert) type I or II tumors were included. The surgery for all the tumors was a transthoracic Ivor–Lewis esophagectomy with intrathoracic esophago-gastrostomy using both open and minimally invasive procedures. The surgical procedure was highly standardized at a high-volume esophageal surgery center, so bias in terms of varying surgical quality was therefore negligible.

Comparison of our survival data with the previously published study has shown that the absolute survival rates are longer than previously reported. To date, the two largest patient populations used to compare perioperative CRT and CT were from the United States, with approximately 7000 patients analyzed through registry analysis.^26,34^ The two studies showed a 5-years survival rate of 37%, which is significantly lower than our results.

Data from retrospective studies in Europe (including our own previous data) from the last years showed rates of 35% to 45%.^19,35,36^ The reason for the improved OS in both the CRT and CT groups compared with the U.S. data cannot be fully explained. Generally, a registry analysis is a very good representation of the quality of care in a country or region. In these studies, however, the previously mentioned reasons may come into effect. These were mixed collectives in which different chemotherapeutic and radiation regimens as well as different surgical techniques were applied.

It is remarkable that our study could achieve survival data at least equal to those in the original CROSS study.^8^ As shown in the original CROSS study, the significant survival advantage in the overall cohort was mediated mainly by the good response of the squamous cell carcinomas co-investigated in the study. In the subgroup of adenocarcinomas, the survival difference reached only the significance cutoff. The reported median OS in the CROSS cohort was 43.2 months compared with 27.1 months in the group without neoadjuvant therapy, which was somewhat shorter than in our analysis with its median OS of 54 months after neoadjuvant CRT.^8^ This is surprising because survival data in controlled randomized trials usually are better than in the real world due to stringent patient selection and very controlled study conditions. The data from our center showed the quality of care in a highly specialized high-volume center, which had less perioperative morbidity and mortality due to optimized perioperative and operative strategies. This could be one reason for the observed longer OS. However, we included only patients with thoracoabdominal esophagectomy. In the CROSS study, approximately 45% of the patients received a transhiatal tumor resection limited to the abdominal approach, which may help to explain the survival difference.

As described earlier, our cohort showed stronger local tumor regression in the CRT group than in the group that had chemotherapy alone, but this was not subsequently reflected in improved OS. Our data for the CRT group very accurately reflected the parameters collected in the CROSS group. In the original CROSS work, a pathologic complete response (pyT0N0) was found in 20% of the adenocarcinomas, the same as in our group. The data collected in the AIO-FLOT study could identify a pCR ypT0 in 17% of the cases, the same as in our collective.^7^ However, we could not achieve the OS of 50 months reported in the AIO-FLOT4 study in our cohort for esophageal adenocarcinomas. The reason for this might have been the mixed population in the study of Al-Batran et al.^7^, which included both gastric and esophageal carcinomas, with only about one third of the patients treated by thoracoabdominal esophagectomy.^7^

The reason why the improved local tumor regression does not result in increased long-term survival is not conclusively understood. Several possible considerations could explain this effect. The relatively weak chemotherapy component in neoadjuvant CRT may not be able to effectively prevent disseminated recurrence compared with full-dose chemotherapy. In addition, complete regression of both primary tumors and locoregional lymphatic metastases is relatively rare. As we could already show in a previous study, especially nodal tumor regression is of predominant relevance as a prognostic factor in the context of tumor regression and long-term survival.^36^

Another possible explanation is the lack of adjuvant therapy after CRT and surgery. The concept of perioperative chemotherapy may have a better chance to target minimal residual tumor disease. The effect of adjuvant immunotherapy after CRT and surgery has led to a doubling of progression-free survival. Data on OS are still pending.^37,38^ However, the effect of postoperative immunotherapy is highly relevant for certain patient groups, and it remains to be seen the extent to which the combination of perioperative chemotherapy and immunotherapy may further improve survival.

A study limitation for discussion was the retrospective character of the study and the incomplete transparency in the selection of the therapy procedure. In retrospect, the decision concerning which patient received which concept cannot be reconstructed. As is evident, patients after chemotherapy have more advanced locoregional tumor stages and more frequent evidence of lymph node metastases. It can of course be argued that these differences are a selection bias and could be compensated for by 1:1 matching. However, based on previous practice, a match must be based on the pathologic tumor-node-metastasis (TNM) staging and not on the pretherapeutic clinical staging. Therefore, because CRT is known to increase tumor regression, matching would not reflect the pretherapeutic tumor stage. It is well known that pretherapeutic staging by endosonography and computed tomography is inaccurate and does not predict well the presence of lymph node metastases in particular because such phenomena as micrometastasis and extracapsular lymph node infiltration are not detectable.^39–42^ For this reason, we intentionally decided against a matching procedure. The patient population was very well comparable concerning the other parameters (e.g., age, gender, comorbidities). Interestingly, we found no difference in survival after stratification for each tumor stage. Therefore, in the end, the therapy method whereby a certain tumor stage was reached seemed to be irrelevant.

The results of the prospective neo-AEGIS and ESOPEC trials, which analyzed the comparison of CT and CRT in a prospective, randomized setting, are awaited with interest.^9–11^ The first results, expected soon, support the retrospective data of the past. The first published data on recurrence-free survival from the neo-AEGIS study do not show clear inferiority of the chemotherapy arm, but it must be noted that besides the FLOT regimen, a variety of chemotherapeutic regimens (ECF/ECX/EOF/EOX) were used, making it difficult to compare the chemotherapy arm with the CRT arm, and long-term survival has not been reported to date.^11^

In conclusion, the question of the ideal therapeutic concept for patients with adenocarcinoma of the esophagus or GEJ I/II cannot be answered definitively, even with our study. No clear survival advantage of one therapy over the other can be identified because treatments with neoadjuvant radiochemotherapy produce significantly less toxicity and are well-tolerated overall. Our recommendation for appropriate patients is that CRT be performed in the neoadjuvant setting instead of perioperative chemotherapy. In addition, the initial data on adjuvant immunotherapy show promising indications, resulting in a new therapeutic option in the adjuvant setting. Chemotherapy alone may be useful for patients with extensive lymph node metastasis, as determined by computed tomography and/or PET-CT, when it is not possible to expand the radiation field to cover all lymph nodes.
